# Trainees' perspective on the best use of supervision-hour in psychiatry training – a qualitative study

**DOI:** 10.1192/bjo.2021.354

**Published:** 2021-06-18

**Authors:** Raja Adnan Ahmed, Mohamed Bader, Mohamed Flensham

**Affiliations:** Aneurin Bevan University Health Board

## Abstract

**Aims:**

This study aims to identify the techniques to improve the quality of the weekly one to one supervision for Psychiatry trainees.

**Method:**

An open-ended online questionnaire was prepared using principles of critical incident technique and distributed among psychiatry trainees working in various deaneries within the UK. The participants were asked to describe an example of a good and a bad supervision experience they had encountered during their training. In addition, participants were also requested to make suggestions to improve the supervision experience. All qualitative data were analysed using the thematic analysis approach, to identify common themes.

**Result:**

A total of 53 trainees working in various deaneries across England and Wales, responded to the questionnaire. The respondents were at a different level of training in psychiatry from CT1-ST6 level. The supervision hour was reported to be useful for clinical case discussions, reflection on difficult cases and situations, pastoral support and wider issues relating to personal and professional development. Trainees appreciated a holistic scope for supervision rather than a narrow discussion of management of cases.

Trainees reported that the supervision hour should be trainee-led and tailored according to their unique learning needs. Participants also saw supervision hour as a safe space where they can receive constructive criticism and feedback on their performance. At times, trust and genuineness were appreciated, as well as the use of an informal tone by the supervisor. An effective supervision leads to trainees feeling valued.

**Conclusion:**

Trainees acknowledged that the supervision hour is an effective tool in psychiatry training. Trainees should get regular, protected and uninterrupted time with consultants for weekly supervisions. Both trainees and trainers need to develop a better understanding of how this supervision experience could be improved and tailored to the individual learning needs of the trainee.

